# Characterization of mature maize (*Zea mays* L.) root system architecture and complexity in a diverse set of Ex-PVP inbreds and hybrids

**DOI:** 10.1186/s40064-015-1187-0

**Published:** 2015-08-18

**Authors:** Andrew L Hauck, Joana Novais, Tony E Grift, Martin O Bohn

**Affiliations:** Department of Crop Sciences, University of Illinois, 1102 S. Goodwin Ave., Urbana, IL 61801 USA; Department of Agricultural and Biological Engineering, University of Illinois, 1304 West Pennsylvania Avenue, Urbana, IL 61801 USA

**Keywords:** Maize, Root, Complexity, Phenotyping

## Abstract

The mature root system is a vital plant organ, which is critical to plant performance. Commercial maize (*Zea mays* L.) breeding has resulted in a steady increase in plant performance over time, along with noticeable changes in above ground vegetative traits, but the corresponding changes in the root system are not presently known. In this study, roughly 2500 core root systems from field trials of a set of 10 diverse elite inbreds formerly protected by Plant Variety Protection plus B73 and Mo17 and the 66 diallel intercrosses among them were evaluated for root traits using high throughput image-based phenotyping. Overall root architecture was modeled by root angle (RA) and stem diameter (SD), while root complexity, the amount of root branching, was quantified using fractal analysis to obtain values for fractal dimension (FD) and fractal abundance (FA). For each trait, per se line effects were highly significant and the most important contributor to trait performance. Mid-parent heterosis and specific combining ability was also highly significant for FD, FA, and RA, while none of the traits showed significant general combining ability. The interaction between the environment and the additive line effect was also significant for all traits. Within the inbred and hybrid generations, FD and FA were highly correlated (r_p_ ≥ 0.74), SD was moderately correlated to FD and FA (0.69 ≥ r_p_ ≥ 0.48), while the correlation between RA and other traits was low (0.13 ≥ r_p_ ≥ −0.40). Inbreds with contrasting effects on complexity and architecture traits were observed, suggesting that root complexity and architecture traits are inherited independently. A more comprehensive understanding of the maize root system and the way it interacts with the environment will be useful for defining adaptation to nutrient acquisition and tolerance to stress from drought and high plant densities, critical factors in the yield gains of modern hybrids.

**Electronic supplementary material:**

The online version of this article (doi:10.1186/s40064-015-1187-0) contains supplementary material, which is available to authorized users.

## Background

Plant root systems are responsible for the vital roles of plant anchorage, absorption of water, and assimilation of nutrients (Lynch 1995). The root system must cope with nutrient availability, moisture deficit and excess, and wind shear on the shoot, along with other abiotic factors such as soil texture and compaction. Biotic stresses on root systems include insect pests, diseases, nematodes, weeds, and competition due to high planting densities, in the case of row crops. A defining feature of root systems is their plasticity and ability to adapt to their environment (Williamson et al. 2001). Root systems respond dynamically to local gradients of moisture and nutrients with directional growth and shape their architecture to explore the heterogeneous soil matrix according to the needs of the plant (Nielsen et al. 1999; Kaeppler et al. 2000; Lopez-Bucio et al. 2003). Root system performance has a major impact on the economics of commercial maize production, given its influence on yield under drought conditions, efficiency of nitrogen fertilizer uptake (Bänziger et al. 2000), and resilience to challenges posed by feeding by the major pest, western corn rootworm (*Diabrotica vergifera vergifera*) (Gray et al. 2009). Silks are 90% water and water status at flowering plays a major role in the fertility of the crop (Troyer 1996). Increased planting densities reduce the available soil moisture per plant, but a significant portion of the improvement in maize yield due to plant breeding can be attributed to gains in performance at high plant densities (Duvick 2005). Furthermore, changes in root system architecture and water acquisition in relation to plant density have shown to be good predictors of the historical elite maize yield (Hammer et al. 2009), highlighting the relevance of understanding the factors that influence root growth, development, and response to external factors. Genetic, environmental, and genotype-by-environment sources of variation for various root traits have been identified for maize (Zhu et al. 2005; Grift et al. 2011; Trachsel et al. 2011). However, there is a scarcity of data on root systems acquired from field experiments, despite the overall importance of the root system for productivity. This is likely a result of the intense labor required to excavate and clean intact root systems on a large scale and the challenge of collecting quantitative data on the numerous fine features of roots within a reasonable time frame. To associate advantageous genetic variation for root characteristics with plant performance, a rapid and accurate means of measuring root traits is needed.

A high throughput image analysis based method for phenotyping stem diameter, root angle, and the fractal dimensions of root systems was recently reported in Grift et al. (2011). Stem diameter and root angle were used to model root system architecture, while estimates of fractal dimension and abundance provide a measure of root system complexity. A root system with a single unbranched root can be described as “simple”, whereas a root system with many highly branched roots can be described as “complex”. Therefore, root complexity is defined in terms of the number of branching points per unit of soil volume, with complexity increasing with branching (Grift et al. 2011). Manually quantifying root branching is impractical at sample sizes considered acceptable for statistical analysis of a typical field study (hundreds or thousands). However, the pattern of branching in root systems, like that of tree branches and certain other biological objects, has the characteristic of self-similarity, in which an object at varying scales provides similar information on the object’s shape or pattern. Furthermore, the frequency of a self-similar pattern at any scale can be quantified by estimation of the fractal dimension (FD). A larger estimate of FD for roots is, therefore, an indication of increased root branching density. Root systems are considered to be approximate fractal objects over a finite range of scales (Tatsumi et al. 1989). The property of self-similarity enables inferences to be made with regard to the whole root system, given a partial observation. This is particularly useful considering the labor required to excavate and clean a complete field grown maize root system with negligible random tissue loss. Root system complexity has been previously evaluated using FD in millet, rye, wheat, pea, peanut (Tatsumi et al. 1989), sorghum (Tatsumi et al. 1989; Masi and Maranville 1998), common bean (Nielsen et al. 1999; Lynch and van Beem 1993), and maize (Tatsumi et al. 1989; Bohn et al. 2006; Grift et al. 2011; Eghball et al. 1993). Wang et al. (2009) related fractal analysis of roots in two rice cultivars to their performance under drought. Transgressive segregation has been observed for root FD in two maize recombinant populations, indicating that the parental inbreds, from which the populations were derived, contain alleles that increase and decrease the trait (Grift et al. 2011).

Maize root system development has been divided into two stages that correspond to embryonic and post-embryonic growth (Feldman 1994). Post-embryonic root development begins approximately 1 week after the primary and seminal roots emerge, as branching of the embryonic roots produces lateral roots that can continue to branch. Lateral roots together with root hairs play an important role in the absorption of nutrients and water by increasing the root’s surface area (Hochholdinger et al. 2004; Gaudin et al. 2011). Approximately 2 weeks after germination the post-embryonic root system becomes prominent, as the coleoptilar node begins giving rise to the crown roots, a type of shoot-borne root that develops from nodes below the soil surface. Brace roots, the second type of shoot-borne roots, develop from nodes above the soil surface several weeks later as the plant matures (Hochholdinger et al. 2004). Crown roots develop from about six compact underground nodes, while the first two or three above ground nodes generate brace roots (Hoppe et al. 1986). Both of these types of roots also exhibit lateral branching, although the brace roots will not branch until they penetrate the soil.

To investigate the contribution of additive and dominant gene action to root system architecture and complexity, the root phenotyping method of Grift et al. (2011) was employed on field experiments using a set of 12 inbreds and their F_1_ hybrids that were described and evaluated for above ground traits in Hauck et al. (2014). This set was chosen in order to evaluate root system characteristics and genetic inheritance within highly improved germplasm. Domestication and improvement of maize significantly altered genetic diversity, gene expression levels, and allele content (Hufford et al. 2012; Jiao et al. 2012). Within the last 40 years, commercial breeding and selection practices have resulted in proprietary maize populations with a wide range of genetic changes relative to less improved germplasm (Hufford et al. 2012). Ten of the inbreds evaluated in Hauck et al. (2014) were developed commercially and formerly protected by plant variety protection (PVP) and all were key founders of modern elite germplasm, based on the most utilized lineages in commercial breeding programs. The generation means design and diallel mating pattern employed enables the estimation of additive effects per se, additive effects in hybrid combination, specific combining ability (dominance), and average mid-parent heterosis.

The objectives of this study were to (1) characterize a selected set of 12 inbreds that were key founders of modern commercial hybrids, 10 of which were formerly protected by PVP, and the 66 hybrids emanating from crossing among the inbreds for root architecture and complexity traits, (2) determine the relative importance of additive and non-additive genetic variation for root system characteristics in this set of elite maize germplasm, and (3) examine relationships between root complexity, root angle, and stalk diameter. Insights on the root system architecture of parental inbred and their F_1_ hybrid progeny can direct the design of future experiments seeking to relate specific root characteristics to agronomic per se and testcross performance using genetic mapping populations derived from the F_1_ hybrids.

## Methods

### Germplasm

In order to assess root traits using germplasm that is more relevant to commercial hybrids, inbreds historically important to commercial breeding programs were identified based on the number of references in Plant Variety Protection certificates and utility patents of proprietary corn inbreds in Mikel and Dudley (2006) and a set of ten Ex-PVP inbreds plus B73 and Mo17 were selected for study. A description of these inbreds is provided in Additional file 1: Table S1 and Hauck et al. (2014), where the 12 inbreds, 66 possible single cross hybrids (without reciprocals), and corresponding F_1_ derived F_2_ populations were evaluated for shoot traits. This representative sample of modern elite germplasm was shown to be genetically and phenotypically diverse (Hauck et al. 2014). Commercial hybrids were evaluated with the experimental F_1_ hybrids as checks. In 2010, sufficient seed was not available for the hybrid entries LH123HT × PHJ40, LH1 × PHJ40, and PHG39 × PHG84. Hybrid B37 × H84 and an additional commercial check were used to replace the missing entries.

### Experimental design

Inbreds and hybrids were evaluated in a generation block design with three replications grown at the University of Illinois Research Educational Center in Urbana-Champaign, IL, in 2007 and 2010 with standard agronomic practices for fertilization and weed management. Inbreds and F_1_ hybrids were blocked separately in the same replication to minimize effects from differences in vigor. Entries were randomized within the generation blocks, and six commercial hybrids were used as checks and randomized with the F_1_ hybrids. The experiment was machine planted with 40 kernels per plot, which were later thinned back to 30 plants per plot. The plot size was 5.33 m long and 0.76 m wide, with one meter alleys, corresponding to a final plant density of approximately 74,000 plants per hectare. Root sampling occurred in the growing season after flowering. Shoot data used for multivariate analysis with root traits was collected from separate three replication trials of the Ex-PVP inbreds and hybrids grown in 2007, 2009, and 2010 at the University of Illinois Research Educational Center in Urbana-Champaign, IL, with the same plot length, planting density, blocking design, and agronomic practices. Shoot data from 2007 and 2008 was reported in Hauck et al. (2014). In 2009 and 2010, four row plots were used instead of single row plots as in 2007, and phenotyping was performed on the center two rows. In 2009 and 2010, another set of three replication trials of the Ex-PVP inbreds and hybrids were grown adjacent to the replications used for collection of shoot data and received the treatment of no nitrogen fertilizer. The effect of the treatment was broadly apparent by the yellowing and reduction in greenness of these trials relative to those with normal fertility, so shoot data was collected on these “low nitrogen” trials.

### Root phenotyping

**Fig. 1:**
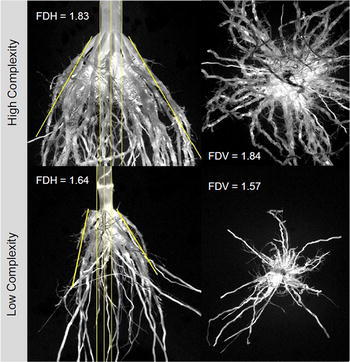
Images display root samples with contrasting complexity, root angle, and stalk diameter. The images have been background subtracted and *gray scaled.*

Root cores (approximately 0.3 m^3^) of five consecutive plants within a plot were dug out and transported to the Agricultural Engineering Farm of the University of Illinois for cleaning. Processed roots were then phenotyped with a custom designed imaging system comprised of a cabinet box with photographic lighting, a light diffusing screen, side view and top view cameras, sample rotation device, and network connection to a desktop computer (Corn Root Imaging Box, CRIB). Details on the imaging system, image processing, image analysis, and control via MatLab^®^ code can be found in Grift et al. (2011). Briefly, the stalk diameter (SD) is measured as the average width, in pixels, of the stalk portion of each of four horizontal side images per sample. Root Angle (RA) was calculated from the same images as the angle of the root cone (Fig. 1). Sample estimates of SD and RA are averages of four perspectives of the root core that differ by 90 degree rotations of the root sample along the stem axis.

The fractal dimension was calculated on images of the underside of the root system samples, which were inverted in the CRIB and viewed from above, using the “box-counting” method and applying equation $log(N_{L} ) = \log \left( K \right) - Dlog(L)$ (Puche and Su 2001). The number of boxes N_L_ with side length L needed to cover a root system in an image is a function of the box side length L and constants D and K. D is the fractal dimension (FD) of the root system and characterizes its branching pattern. For 2D images, values for FD vary between 1 (“single un-branched root”) and 2 (“highly branched system”). The logarithm of K is called fractal abundance (FA) and indicates to what degree root systems explore the soil space, i.e., the number of root branches present in a given soil volume (Walker et al. 2004).

The data was filtered for outliers attributable to difficulties in image processing, then averaged to produce mean values per plant. Subsampling the root system with multiple images provides better estimates for RA and SD by reducing the potential for bias from sampling 3D root structure from a single 2D perspective. The redundancy of information obtained from different perspectives of the root system samples was assessed with Pearson correlation coefficients. The correlation between one of the four estimates of RA per sample with the sample average’s RA was r_p_ ≈ 0.88 across entry classes. Correlations between individual SD estimates and the sample average were also strong, r_p_ ≈ 0.93 across entry classes. FD and FA data from images of root system undersides from the same sample were nearly perfectly correlated (data not shown), so only one image from the vertical axis was used for calculation of root sample FD and FA. Estimates of FD derived from orthogonal (90 degree) horizontal perspective images of root samples were highly correlated r_p_ ≥ 0.90 and the correlation between underside and side perspectives was also high for inbreds (r_p_ = 0.77), F_1_s (r_p_ = 0.90), and check (r_p_ = 0.93) entries. Since the fractal estimates from different perspectives are highly correlated and the side profile images contain the stem, which may reduce the accuracy of FD estimates, estimates of FD from the underside profile of root cores were used for analysis. Statistical analyses were conducted using plot means of the root traits. Root and shoot data was adjusted for maturity based on days to silking and converted to Z scores prior to multivariate analysis.

### Statistical analysis

A modified version of Eberhart and Gardner’s (1966) general model was used to estimate genetic effects. Due to the number and type of generations included in this study, Eberhart and Gardner’s line heterosis and specific heterosis effects are equivalent to general combining ability (GCA) and Specific Combining Ability (SCA). Since the inbred parents of the F_1_ hybrids are a selected set rather than a segregating population, genetic parameters are fixed effects. Overall significance of a genetic parameter in the model, such as the additive effects per se, is indication that variance due to that parameter is important for determining the trait phenotype within the set of material tested. The overall model accounting for genetic effects and the experimental design was:

1 $$\begin{aligned}
Y_{ijklm} &= \mu + {\rm e}_{\rm i} + {\rm r}_{{\rm ij}} + {\rm b}_{\rm k}
+\left[ {\rm a}_{\rm l} \times\beta +
{\rm a}_{\rm m} \times \beta \right]
+ \left[
{{\rm g}}_{\rm l} \times \gamma +{\rm g}_{{\rm m}}
\times \gamma + {\rm s}_{\rm lm} \times \delta \right]\\
&\quad+ {\text{eb}}_{\text{ik}} + {\text{rb}}_{\text{ijk}} +
{\text{d}}_{\text{ilm}} + {\text{c}}_{\text{n}} +
{\text{ec}}_{\text{in}} + \varepsilon_{\text{ijklmn}}
\end{aligned}$$

for each trait plot mean *Y* with intercept µ and the following effects with indexes for location (i), replication (j), entry class (k), genotypic effects (l, m), and the plot (n): e_i_ = ith environment, i = 1, 2; r_ij_ = jth replication nested in the ith environment, j = 1, 2, 3; b_k_ = effects accounting for the means of inbred, F_1_, and check classes of entries, k = 1, 2, 3; a_l_|a_m_ = effect of the lth and mth line per se (additive effect), l, m = 1, 2, …, 12; g_l_|g_m_ = general combining ability of the lth and mth line; s_lm_ = specific combining ability of the cross of the lth and mth lines; eb_ik_ = interaction of the ith environment with the kth entry class effect; rb_ijk_ = interaction of the jth replication nested in the ith environment with the kth entry class; d_ilm_ = interaction of the genetic effects a_l_, a_m_ with the ith environment; c_n_ = effect of the nth check entry; ec_in_ = interaction of the nth check entry with the ith environment; ε_ijklmn_ = residual error.

The design parameters were specified as

β = 1.00 for the Ith parent and β = 0.50 for the cross between the lth and mth inbreds in the F_1_ hybrids block; β = 0 for the check entries. γ = 1.00 for cross between the lth and mth inbreds in the F_1_ hybrids block and γ = 0 for the check and parent entry classes; δ = 1.00 for the cross between the lth and mth inbreds in the F1 hybrids blocks and δ = 0 for the check and parent entry classes;

Note that the entry class effects, b_k_, can be partitioned into two orthogonal contrasts, each with one degree of freedom; viz,

Mean (mid-parent) heterosis = (F_1_ blocks mean − Parents blocks mean),

Mean difference between diallel F_1_s and commercial hybrid checks = (F_1_s mean − Checks mean).

Statistical analyses were conducted with SAS/STAT software, Version 9.2 of the SAS System, using the CORR, MEANS, MIXED, MULTEST, FASTCLUS, and CANDISC procedures (SAS Institute 2008). Phenotypic information that was unavailable for certain plots was entered as missing data, resulting in an unbalanced design. The MIXED procedure to conduct Type III ANOVAs with all factors and interactions as fixed effects using a manually generated design matrix, consistent with the parameterization in (1), with the usual ‘sum to zero’ restrictions on the parameters to ensure estimability. Contrast statements were used to determine the overall significance of genetic effects, and estimate statements provided genetic effect estimates for each line or cross, means of the entry classes, the magnitude of differences between Ex-PVP hybrids and checks, and mean heterosis, estimated as the mean of the F_1_s minus the mean of the inbreds. The significances of reported model parameters and genetic effects were adjusted using the adaptive false discovery rate (Benjamini and Hochberg 2000). Pearson correlation coefficients (r_p_) were calculated among root characteristics using plot means for each entry class.

## Results

### Phenotypic means

Parental inbreds had smaller mean FD, FA, and RA trait values (q < 0.001) and narrower trait ranges than F_1_ hybrids, but the standard deviations for each trait were similar across generations (Table 1). Stalk diameter means among the entry classes were not significantly different. Inbred RA means were about fourteen degrees narrower than that of the F_1_s. Values for RA ranged from 37 to 86 degrees among inbreds, 46 to 102 degrees across F_1_ hybrids, and 52 to 85 degrees among the checks. Hybrid RA may be increased relative to inbred RA due to the greater frequency of higher nodes of brace roots contributing to hybrid root systems. However, the distribution of hybrid RA means reflects substantial segregation for this trait. Reduced root branching and soil exploration of inbreds vs. hybrids, as indicated by lower FD and FA scores, is consistent with performance reduction associated with inbreeding depression. Overall, parental root structure was less complex and the root systems narrower than hybrid root cores, but inbred stalks were as thick as hybrid stalks. The root structure of Ex-PVP F_1_ hybrids appear to be similar to that of the commercial checks investigated.

**Table 1:** Mean, standard deviations, and minimum and maximum values for maize root traits observed in 2007 and 2010 Urbana, IL

Trait^a^	Mean ± standard deviation			Minimum–maximum		
	Parents	F_1_s	Checks^b^	Parents	F_1_s	Checks^b^
	*N* = 12	*N* = 66	*N* = 5			
FD	1.756 ± 0.052	1.794 ± 0.048	1.803 ± 0.046	1.641–1.847	1.672–1.902	1.722–1.875
FA	11.04 ± 9.80	11.35 ± 10.03	11.33 ± 9.89	10.03–11.52	10.44–11.81	10.76–11.74
RA (°)	59.7 ± 11.3	74.2 ± 10.2	69.5 ± 8.5	37.2–86.5	46.1–102.5	52.8–84.9
SD (pixel)	116.5 ± 36.2	118.8 ± 29.2	124.6 ± 30.7	54.5–213.6	47.6–225.2	66.8–191.6

^a^ *FD* fractal dimension, *Log(FA)* fractal abundance, *RA* root angle, *SD* stalk diameter. Plot means (*N* = 72, 387, 30) were used to obtain the values for the 12 “Parents”, 66 “F1 hybrids”, and 5 “Check hybrids” entries.

^b^Only data for the five checks present for both environments were included in calculations.

### Phenotypic correlations

Across entry classes, FD was significantly correlated with FA (r_p_ ≥ 0.74, Table 2). No significant relationship between fractal based root characteristics and RA were detected, besides a significant association between FD and RA for parental inbreds (r_p_ = −0.40). Stalk diameter showed moderately sized positive correlation coefficients with FD and FA (r_p_ = 0.48) but small correlation coefficients with RA. The high correlation between FD and FA suggests a relationship between the level of branching and total observed root length, while their moderate correlation with SD is likely a consequence of larger plants having larger root systems.

**Table 2:** Phenotypic correlation coefficients among root traits by entry class

Entry class	*N* ^a^		Pearson correlation coefficients			
			FD	FA	RA	SD
Parent	72	FD	1			
		FA	0.74***	1		
		RA	−0.40**	0.06	1	
		SD	0.48***	0.54***	0.02	1
F_1_	386	FD	1			
		FA	0.88***	1		
		RA	−0.05	0.13*	1	
		SD	0.61***	0.61***	−0.13**	1
Check	45	FD	1			
		FA	0.88***	1		
		RA	−0.10	−0.09	1	
		SD	0.69***	0.68***	−0.33*	1

*FD* fractal dimension, *FA* fractal abundance, *RA* root angle and *STD* stalk diameter.

***, **, * Correlation coefficients are significant at the 0.0001, 0.001, and 0.05 unadjusted probability levels, respectively.

^a^Number of plot means per entry class.

### Model effects

The additive effect of the parental inbreds per se was highly significant across traits, but general combining ability was not significant for any of the phenotypes (Table 3). Specific combining ability was significant (q ≤ 0.01) for FD and highly significant (q ≤ 0.001) for FA and RA (q ≤ 0.001). Highly significant heterosis was observed for FD, FA, and RA, but not for SD. The line × environment interactions were highly significant for all root phenotypes except SD. Highly significant variation among checks was observed for FD, FA, and RA. F_1_ hybrids differed from check hybrids for FA (q ≤ 0.05) and RA (q ≤ 0.001), but not for FD and SD. The effect of the environment was highly significant for FD, FA, and SD, but not for RA. The magnitude of the environmental effects observed for FA was similar to mean heterosis for FA, more than twice mean heterosis for FD, and about one standard deviation of the entry class means for SD. Ex-PVP F_1_s differed from the checks for mean FA and RA, but not FD or SD.

**Table 3:** Model parameter F-values, significance level, and estimates of environmental differences, heterosis, and differences between F1 and check hybrids for FD, FA, RA, and SD

		F value			
Effect	DF	FD	FA	RA	SD
ENV	1	512.27***	283.89***	0.00	89.99***
Reps (ENV)	4	0.38	2.19	2.10	1.28
Effect of lines per se	11	12.80***	10.14***	11.54***	4.94***
GCA	11	0.93	1.23	1.56	1.57
SCA	54	1.76**	1.98***	2.39***	0.97
Checks	7	3.89**	3.87**	6.70***	1.71
Line × ENV	11	4.72***	2.95**	3.30**	2.44*
Heterosis × ENV	1	0.11	23.20***	10.54**	0.30
F1 vs. Checks × ENV	1	0.04	2.13	2.78	1.33
Checks × ENV	4	4.14*	3.96*	2.34	0.71
Residual covariance	395	4.88 × 10^−4^	1.177 × 10^8^	49.00	568.99

Estimate		Estimate [standard error]			
2007 vs. 2010	1	−0.074***	10.2116***	0.1	−32.6***
		[0.003]	[7.3873]	[1.0]	[3.6]
Mean heterosis, (F_1_s vs. Inbreds)	1	0.038***	10.0434***	14.5***	2.3
		[0.003]	[7.2410]	[0.9]	[3.1]
F_1_s vs. Checks	1	−0.004	8.5524*	5.2***	−5.8
		[0.004]	[7.5104]	[1.2]	[4.0]

*ENV* environment, *Reps* replications, *FD* fractal dimension, *FA* fractal abundance, *RA* root angle, *SD* stalk diameter, *GCA* general combining ability and *SCA* specific combining ability.

***,**,* Indicate significance of effects at probability levels q ≤ 0.001, q ≤ 0.01, and q ≤ 0.05, respectively.

An overview of the temperature and precipitation in the two growing seasons is provided as supplementary information. Both environments had similar average temperatures, but the period of plant vegetative growth in the 2007 environment experienced less rainfall, more moisture evaporation, and half as many days with rain compared with the 2010 environment (Additional file 1: Table S2). Root systems were much less complex and had smaller stalks in the 2007 environment (Table 3), which had a large average water deficit. In the 2010 environment, the amount of rainfall and evaporation was balanced. Data collected from trials conducted in 2007 and 2010 for shoot phenotyping suggests a more nuanced interpretation, however. Based on contrasts of least square means of the inbreds and hybrids, plant height was greater in 2010 by 21.5 cm (±1.4 cm) and flowering time was delayed by 12.2–12.5 days (±0.2 days to anthesis/silking) compared with 2007. Despite the more favorable moisture conditions in 2010, the grain weight per ear was 31.2 g (±2.4 g) greater in 2007. Mean heterosis was also significantly different between the environments for FA and RA (q ≤ 0.01), with increased heterosis observed in 2010. Since the material evaluated is a selected set of germplasm, broad sense repeatabilities were calculated for the inbreds and F_1_s instead of heritabilities and provided in Additional file 1: Table S3. Repeatabilities for FD, FA, and RA among the inbreds were high (0.89, 0.87, 0.89), and greater than those of the F_1_s (0.67, 0.74, 0.78). Repeatabilities for SD among the inbreds and F_1_s were moderate (0.50, 0.39), but the 90% confidence intervals were large, particularly for the inbreds. This variation is interpreted to be related to within-plot plant to plant variation for plant size based on observations during sampling.

The additive effects of the parental inbreds are provided in Table 4. The twelve inbreds were grouped based on the size and direction of their root trait effects. LH123HT, PHZ51, Mo17, PHG39, and PHG84 each had positive additive effects for root traits when significant. LH123HT and PHZ51 had average FD and above average FA, while PHZ51 was also characterized by a wide root angle and large stalks. Mo17 was only above average for FD, while PHG39 and PHG84 contributed strong positive effects for FD and FA, but are otherwise average for the root architecture traits. B73 stands out from the other inbreds; it has the largest positive effect on FD, average FA. the most narrow root angle, and thick stalks. LH1 and LH82 have the largest negative effects for FD, below average FA, as well as strong positive effects for RA. PHG47, PHJ40, and PH207 have negative effect estimates for all significant estimates of root traits. The effect estimates of FA and SD for PHJ40 were exceptionally below average compared with the other parents. PHG35 is estimated to have average characteristics for all root traits in this set of germplasm. There is no obvious association of the heterotic groups with particular root traits, but earlier maturity lines in the set, including PHJ40, LH82, PHG47, and PH207, tend to have negative effect estimates for FD, FA, and SD. This was also observed for shoot traits in Hauck et al. (2014) and is likely related to plant size at flowering. LH1 has the least complex root system and above average maturity, but is also shorter than average. Based on F_1_ entry least square means from 2 years, the correlation of FD, FA, and SD with plant height was moderate (r_p_ = 0.66, 0.71, 0.49), while the correlation with either days to anthesis or days to flowering was high (r_p_ ≈ 0.84, 0.88, 0.72). Plant height is moderately correlated with flowering time (r_p_ = 0.65), while the correlation of RA with plant height and flowering time was low (r_p_ = 0.11, 0.21). Overall, these observations are consistent with root and shoot development being coordinated.

**Table 4:** Genetic effects of the parents per se estimated from the general model for FD, FA, RA, and SD root traits

Inbred	Pool	FD	FA	RA	SD
Positive additive effects for root traits					
LH123HT	N	0.016	19,803***	2.6	17.4
PHZ51	N	0.003	13,671**	13.6***	38.6***
MO17	N	0.022*	7,505	2.7	1.0
PHG39	S	0.033**	11,046*	0.1	−7.9
PHG84	N	0.029**	12,864*	−2.5	4.8
Positive FD and SD, negative RA					
B73	S	0.054***	1,877	−19.2***	24.6*
Negative FD and FA, positive RA					
LH1	S	−0.051***	−13,122*	13.0***	−5.5
LH82	N	−0.043***	−9,488^○^	9.1**	−18.6
Negative additive effects for root traits					
PHG47	N	−0.029**	−11,585*	−5.1	−4.4
PHJ40	S	−0.023*	−28,702***	−9.1**	−40.5***
PH207	N	0.006	−5,983	−9.1**	−8.8
Neutral root trait effects					
PHG35	N	0.000	2,115	3.9	−2.5
SE^a^		0.009	4,240	2.7	9.3

*Pool* heterotic pool, *S* stiff stalk synthetic, *N* non-stiff stalk, *FD*, fractal dimension, *FA* fractal abundance, *RA* root angle and *SD* stalk diameter.

^a^Standard error for genetic effects. False discovery rate significances are shown as *** q ≤ 0.001; ** q ≤ 0.01; * q ≤ 0.05; ^○^ q ≤ 0.1.

**Fig. 2:**
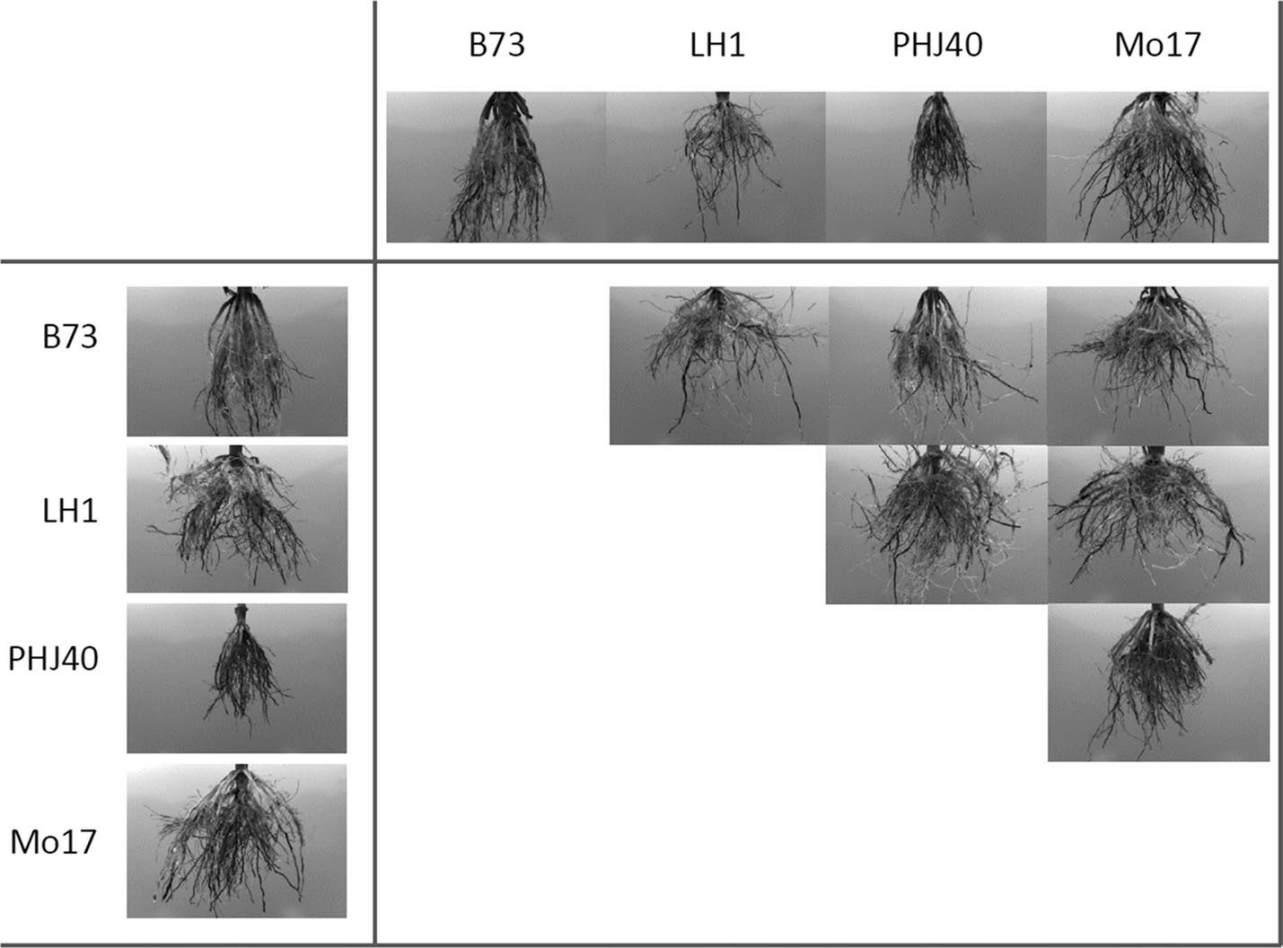
Root samples of maize inbreds B73, LH1, PHJ40, and Mo17 and of hybrids obtained from their crosses. Roots of inbreds displayed in the first column and row, respectively, were obtained from plants sampled from different field plots.

Least square means of the five highest and lowest performing F_1_ hybrids for FD and RA traits are shown in Table 5. The hybrids with the highest and lowest performance for root traits generally agrees with expectations derived from Table 4, which is expected, given the importance of additive effects for the phenotypes. There are some notable deviations, however. For example, inbreds LH1 and LH82 had the strongest negative effects for FD, but there were nine hybrids with lower FD estimates. PHG47 and PHG84 were estimated to have non-significant effects for RA compared with the mean of the set, but their cross had the 4th widest root angle. This is consistent with the detection of significant SCA among the hybrids for root traits and evidence for the role of dominance in root trait expression. A summary overview of hybrid performance rankings, genetic effect magnitudes, F_1_ means and mid-parent values for root traits is provided in Additional file 1: Figs. S1–S4. The general additivity of root traits can be seen in comparisons of inbred and hybrid images, where the F_1_s have mid-parent characteristics (Fig. 2). The specific dominance detected in the cross of B73 by LH1 for root angle can also be seen; the F_1_ has a wider root angle more closely resembling LH1.

**Table 5:** Least square means of experimental F_1_ hybrids with the highest and lowest performance for fractal estimated root complexity

Hybrid	Cross^a^	FD	FA	RA	SD
Highest fractal dimension					
B73 × PHG84	SN	1.849	110,055	69.8	133.2
B73 × PHG39	SS	1.842	89,725	65.7	136.4
B73 × PHZ51	SN	1.841	104,597	69.1	129.2
MO17 × PHG39	NS	1.835	103,815	81.1	130.1
PHG39 × PHZ51	SN	1.835	108,746	83.2	120.2
Lowest fractal dimension					
LH1 × PHJ40	SS	1.731	58,682	72.8	94.8
LH82 × PHJ40	NS	1.756	64,301	69.4	98.7
LH1 × PHG47	SN	1.762	76,099	81.8	117.3
LH82 × PHG47	NN	1.765	75,018	76.7	100.6
PHG35 × PHJ40	NS	1.768	67,873	69.6	94.9
Widest root angle					
LH1 × PHZ51	SN	1.786	91,661	89.1	118.7
PHG47 × PHZ51	NN	1.779	90,545	88.5	118.0
LH1 × LH123HT	SN	1.786	86,378	86.4	109.4
PHG47 × PHG84	NN	1.806	103,906	85.7	98.8
LH82 × PHZ51	NN	1.773	87,003	84.7	106.5
Most narrow root angle					
LH82 × PH207	NN	1.775	74,606	62.9	122.2
B73 × PHG35	SN	1.813	86,539	61.8	126.1
B73 × PHJ40	SS	1.800	70,524	60.7	117.7
PH207 × PHJ40	NS	1.782	71,989	57.9	118.6
B73 × PH207	SN	1.785	76,911	53.3	131.8
Standard error of L.S. means		±0.010	±4,782	±3.0	±9.9
F_1_ mean		1.795	85,265	74.2	118.8

*FD* fractal dimension, *FA* fractal abundance, *RA* root angle and *SD* stalk diameter.

^a^Cross indicates whether the hybrid is an intra-pool or an inter-pool cross, parental inbreds are coded as S, Stiff stalk synthetic, N *Non*-stiff stalk

### Multivariate analysis of root and shoot data

**Fig. 3:**
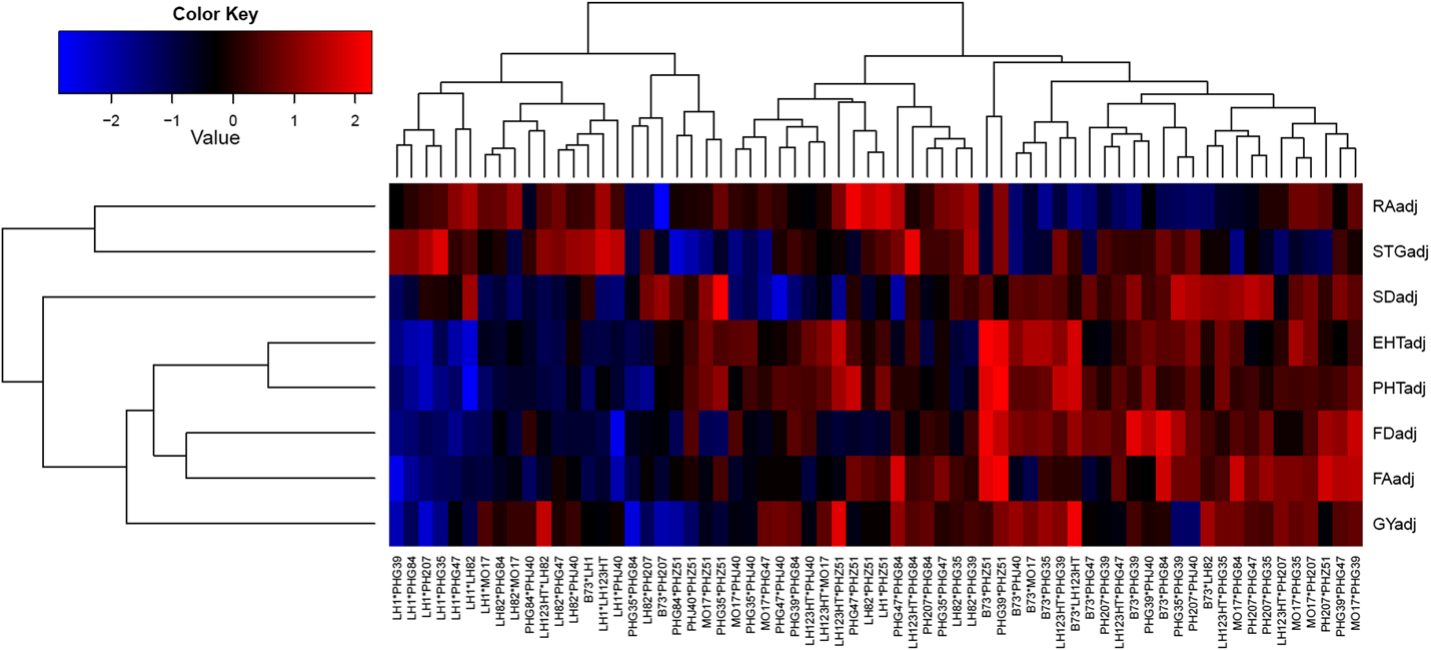
Hierarchical cluster analysis of 66 F_1_ hybrids based on Euclidian phenotypic distances. The heat map represents performance of each hybrid within each sub cluster for maturity adjusted agronomic traits (*EHT* ear height, *PHT* plant height, *GY* grain yield, *STG* stay green) and root characteristics (*FA* fractal abundance, *FD* fractal dimension, *RA* root angle and *SD* stem diameter).

Root and shoot phenotypes were collected from separate experimental trials grown in 2007 and 2010, then combined into individual estimates for each hybrid as least square means. Principle components and loadings from the correlation matrix of root fractal dimension, fractal abundance, angle, stem diameter, ear height, plant height, stay green score, and five ear samples of grain weight for the 66 experimental hybrids tested are presented in Table 6. The first two components explain 86% of the variation. Based on the loadings, staygreen and root angle are distinguished from height, fractal, stem width, and grain yield traits by component one, while component two primarily contrasts stalk diameter with grain yield and root angle. These contrasts are evident in the three major groups of roughly equal size that result from hierarchical cluster analysis of the F_1_ phenotypes based on Euclidian phenotypic distances (Fig. 3). Ten half-sibs of LH1 cluster together and tend to have wider root angles, above average staygreen, and below average height and root complexity. Similarly, another cluster containing nine B73 half sibs have below average root angle and above average height and root complexity traits. It is worth noting that there is no obvious association with the estimates of vegetative phenotypes and grain yield. In this set of material, fractal abundance, fractal dimension, stem diameter, tended to increase with plant height, ear height, and grain yield, while entries with lower values for these traits had wider roots and stayed green longer and were related to LH1.

**Table 6:** Loadings of Principal Components (PC) 1 and 2 of the correlation matrix of root and shoot phenotypes observed in 2007 and 2010

	PC1	PC2
Eigen value	2.31	0.19
Variance explained (%)	67.0	19.0
Stay green	0.38	−0.06
Root angle	0.33	0.38
Grain yield	−0.22	0.62
Stem diameter	−0.25	−0.63
Fractal abundance	−0.37	0.05
Plant height	−0.40	0.21
Fractal dimension	−0.41	−0.09
Ear height	−0.41	0.15

The least square means estimates of root traits from these trials were then scaled to mean 0 and combined with least square means of shoot data collected in 2009 and 2010 under normal nitrogen fertility and fields with no nitrogen added. Shoot phenotypes, including grain yield, plant barrenness, kernel test weight, and SPAD meter reading, were adjusted based on maturity within the nitrogen treatment and estimates of root traits were adjusted for the maturity observed in 2009 and 2010 under normal fertility. The correlation matrices of root and shoot phenotypes for the two nitrogen treatments were analyzed by principal component analysis. For both treatments, there were three eigenvalues greater than one that explained a total of ~80% of the variance (data not shown). A plot comparing the first principal component of the two treatments shows little change in the common variance and resembles results observed from analysis of 2007 and 2010 data (Additional file 1: Fig. S5A). In particular, the first component primarily distinguishes root angle and staygreen from fractal abundance, fractal dimension, plant height, and ear height, along with stem diameter and grain yield, to a lesser extent. Test weight groups near root angle and staygreen. The correlation between SPAD meter reading and the component is 0.22 under normal fertility and 0.01 at low N, which explains the deviation of the score. The second component discriminates the treatments more. Under normal fertility, grain yield, SPAD, RA, and FA are contrasted with barrenness and test weight, while under low fertility barrenness and SPAD are contrasted with stay green (Additional file 1: Fig. S5B). Presumably, barrenness resulted in higher leaf nitrogen due to absence of a sink and was, therefore, detected by SPAD. In summary, estimates of root traits for hybrids examined at normal fertility in 2007 and 2010 displayed a very similar pattern of variance when paired with shoot data at normal fertility from either 2007 and 2010, 2009 and 2010, or low fertility in 2009 and 2010.

## Discussion

### Methodological justification

This study employed a high-throughput method to analyze the root architecture of mature field grown inbreds and hybrids that is a sample of the genetic diversity of modern elite germplasm. Core root systems of about 2,500 plants were dug, cleaned, and phenotyped using an image-based approach with high repeatability for FD, FA, and RA. The limitations of this experimental methodology include: (1) root systems are evaluated for a single developmental stage only, (2) the deep portion of the root system is not sampled, and (3) processing of each root system for imaging incurs some significant random tissue damage and loss to the root core and adversely affects the fine root structure. Despite these limitations, we were able to study the genetic basis of root architecture in our set of commercially relevant germplasm.

Due to the large number of genotypes in this experiment, we were not able to evaluate root structures at multiple developmental stages. Therefore, we decided to determine root traits only after flowering, with the assumption that the architecture of roots harvested post-anthesis integrates the response of plants to all environment-specific soil properties (e.g., soil moisture and temperature changes) and growing conditions (Füllner et al. 2012). Given the correlation between maturity and root traits, phenotyping before all plants have flowered could result in un-equal comparisons. In addition, it is not feasible in a large-scale field experiment to recover complete root systems. Instead we focused our efforts on a 0.3 m^3^ root core volume. This sampling method utilizes a large proportion of the root system for estimation of complexity based on previous observations by Dwyer et al. (1996), who estimated that 90% of total maize root biomass is located in the top 0.3 m soil profile. Others have also found that most of the maize root biomass is located near the surface (Amos and Walters 2006), indicating that root core samples represent the major fraction of the total root system.

**Fig. 4:**
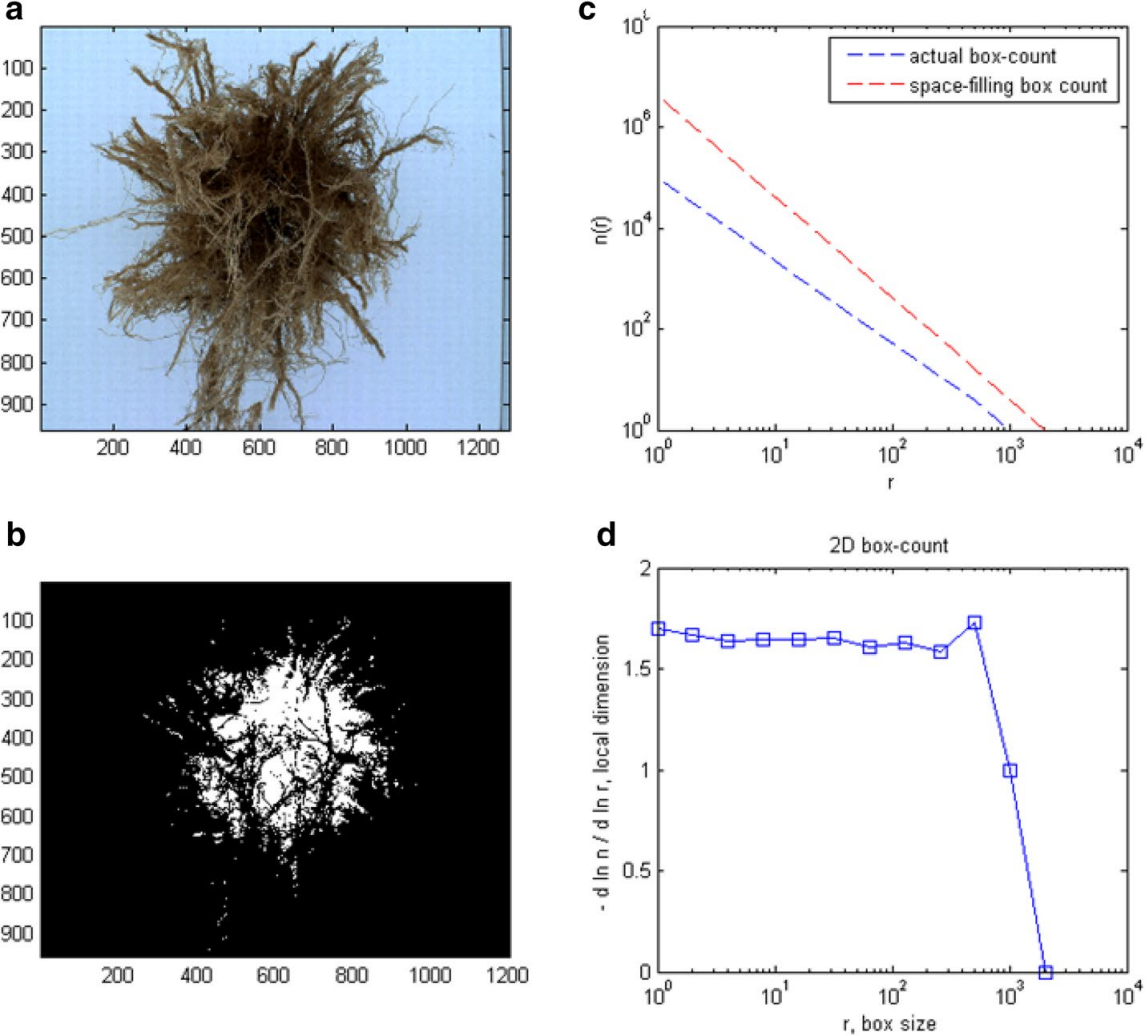
Vertical image of a hybrid maize root system. **b** Color image in (**a**) was processed into a binary black and white image. **c** The image in (**b**) was used for calculating the box dimension. The log–log graph relates box side length *r* to the number of boxes *N* needed to cover the root shown in (**b**). The slope of the “space filling box count” *line* is the box dimension of the root in image (**b**). **d** This plot shows the local box dimensions for image (**b**). This graph indicates that *N* and *r* are related by the power law $N = r^{ - Dim}$ within the scale range of 1–256 pixels. All images and graphs were produced using the public Matlab program “boxcount.m”.

While this study did not explore the question of whether maize roots can be conclusively classified as fractal objects, our assumption that roots are fractal-like helped us to overcome limitations caused by the analysis of partial root systems. If roots are indeed fractals, loss of fine structure during the washing processes will not alter the fractal dimension estimate of a root system. In addition, the use of fractals facilitates extrapolation of root complexity to the unobserved portions of the root system. The box counting method is the standard approach applied to determine the fractal dimension of root systems. Box counting algorithms tally the number of boxes *N* of a given side length *s* needed to cover an object in the image. If this object is a fractal, *N* and *s* are related by the power law $N = s^{ - Dim}$, where *Dim* is the “box dimension” of the object and plotting $\log N$ versus $\log s$ results in points on a straight line with *Dim* being the slope of this line (Fig. 4c). With experimental data, *Dim* is commonly estimated by fitting the linear model $\log N = - Dim \log s$ using the least-square approach to the data (Clauset et al. 2009). However, least-square fits do not provide information whether the data was indeed sampled from a power-law distribution. Clauset et al. (2009) developed a goodness-of-fit test for large data sets based on the Kolmogorov–Smirnov statistic and likelihood ratios. How these goodness-of-fit tests function with small data sets, like in our case, where each image provides only a set of ten data points using the box counting method to determine fractal dimensions, is yet unknown.

We observed FD values for hybrid roots ranging from 1.67 to 1.90 (Table 1) over a finite scale range of 1–512 pixels. This scale range spans 2.7 orders of magnitude (Additional file 1: Table S4). Halley et al. (2004) discussed in their review “Uses and abuses of fractal methodology in ecology” the effects of too few scales on estimating fractal dimension, specifically in cases where self-similarity is observed only for small scale ranges. In a Web of Science survey of papers on fractals published in ecological journals, Halley et al. (2004) found that studies spanning more than three orders of magnitude are rare. In previous root studies, the order of magnitude varied between 0.7 (wheat, Manschadi et al. (2007)) and 2.5 (rice, Wang et al. (2009)) with our study exceeding the previous scale ranges (Additional file 1: Table S4) and supporting the hypothesis that roots follow self-similar properties over a wide range of possible scales. If roots can be treated as fractal objects, we expect that images of non-overlapping parts of a root system show similar fractal dimension estimates. In accordance with this expectation, correlation coefficients between FD values obtained from different views of the same root system were highly significant (p ≤ 0.001), as noted in the materials and methods. This supports the premise that root systems can be treated as fractal-like objects, at least over the scale range used in our experiment (Fitter 2002).

### Interpretation of results

Grift et al. (2011) evaluated 200 recombinant inbred lines (RILs) from the cross B73 × CML333 test crossed with PHZ51 and reported a maximum value of FD of 1.878, which is similar to the maximum observed F_1_ hybrid FD in our experiment. However, the average FD value in our study is on the low end of the FD distribution in Grift et al. (2011). This is likely a result of a combination of several factors. Our study included inbreds with low root complexity, which extended the minimum observed F_1_ hybrid FD values by another tenth of the FD scale below those reported in Grift et al. (2011). Among the Ex-PVP lines evaluated in our study, B73 had the greatest effect on FD and PHZ51 showed the second largest effect of FA, indicating the germplasm tested in Grift et al. (2011) can be expected to have above average complexity relative to our set. The Urbana 2007 test environment resulted in lower population-wide root FD estimates than the Urbana 2010 test environment. The 2007 environment was characterized by drought stress, which reduces accumulation of root and shoot biomass (Bänziger et al. 2000), though reproductive biomass of the grain was greater in 2007. The significantly lower mean FD and FA values observed in 2007 are suggestive of reduced root biomass, if FA is related to root length and greater amounts of root branching are associated with increased root biomass. The detection of highly significant additive and non-additive effects for FD, FA, RA, and SD indicates the presence of genetic variation for root complexity and architecture, as defined, in Ex-PVP germplasm. The highly significant additive effect-by-environment interaction for the root traits is also of interest, as it indicates variation in how inbreds responded to the two contrasting environments with regard to root system architecture and complexity.

The means for RA in our study were similar to those reported by Grift et al. (2011), but the range of observations extended slightly below 40° and above 100 degrees. The environments had no effect on the generation means of RA. This is interesting considering the strong influence of the environment on the other root architecture features. Root angle may be determined relatively early during plant growth, specifically as a response to plant competition within the row, and be less responsive to later environmental conditions. Factors such as differences in soil texture and fertility that were not tested in our study may influence root angles on a population-wide basis, though Troyer (1996) notes that hybrid by soil type interactions on performance of commercial germplasm were not frequently observed. Trachsel et al. (2011) rated brace root angle visually on a scale of 1–9 and crown root angle on a scale of 3–9 using dug and cleaned root cores from 98 B73 × Mo17 derived RILs, a subset of the inter-mated B73 × Mo17 (IBM) population, evaluated in three environments. They reported that the most repeatable root architecture trait for the IBM population in their study was crown root angle at 67%. As our method for calculating RA does not involve removing brace roots, it can be understood more as a brace root angle measurement, though lines and hybrids are known to differ in the number of nodes they form brace roots from, which can influence the root angle calculation. Our RA measure is one of root topology, regardless of whether crown or brace roots form the outer perimeter of a root system. Across a larger set of RILs chosen for root architecture diversity, the Spearman-rank correlation between brace and crown root angle in Trachsel et al. (2011) was about 0.5. While brace root angle may differ from crown root angle, brace roots comprise the bulk of post-embryonic root biomass at maturity (Fig. 1), so root angle measurements based on them may be more related to other traits of interest.

The distribution of plot means for SD was similar across generations and significant heterosis was not observed for this trait. This was unexpected, given the differences in plant size of hybrids compared with inbreds. PHZ51 and B73 contributed a large positive effect on SD, while only the earliest inbred, PHJ40, reduced SD significantly. Significant variation for SD among checks was not observed, though they exhibited significant variation for FD, FA, and RA. Based on our experience, estimates of SD are likely to be more sensitive to variability in plant spacing within a plot. Plants that are closer together or further apart than the target density are subject to increased or decreased stress from plant competition, which has a strong influence on plant size. Due to the shade avoidance response, higher plant densities have been associated with increased plant height and reduced stem diameter in maize (Sangoi et al. 2002). Density stress may also significantly affect FD, FA, and RA, though the repeatabilities for these traits was much higher than for SD. This is a particularly interesting avenue of future research, given the importance of increased plant density stress tolerance in historical improvements of maize grain yield (Duvick 2005). Stalk diameter was moderately positively correlated (0.68 ≥ r_p_ ≥ 0.48) with FD and FA among the parents, F_1_s, and checks, indicating that larger stemmed plants tended to have more complex root systems. As discussed previously, fractal based estimation of root branching should not be unduly biased by the overall size of the root system. Since proportional plant growth for some range of shoot to root ratios is a favorable adaptation for grain yield, the positive correlation between stem diameter and root complexity in our set of material might be expected if these traits are predictive of shoot and root mass. Evaluating the performance of elite hybrids for root system architecture and complexity under density and fertility stress as it relates to yield is also a logical extension of this work. A comparison of root effects to shoot data from normal and reduced fertility environments did not reveal any strong association with low N performance and common variance among phenotypes appeared to be related to pedigree, as might be expected in evaluation of a diallel.

### Role of additive and non-additive variation in root architecture and complexity

Comparable studies investigating heterosis in maize for mature post-embryonic root architecture and complexity in field experiments appear absent. Experiments reporting data on the structure of the maize post-embryonic root system in general are rare, so this work addresses a topic with sparse information. Highly significant mean heterosis and SCA, a measure of dominance plus inter-gametic epistasis, was observed for each trait except stalk diameter. In contrast to the additive effect of the lines, significant GCA was not observed for any trait, indicating that the per se performance of the parents is sufficiently predictive of their performance in hybrid combination. The correlation structure of the root phenotypes was evident when the parents were grouped into contrasting classes based on additive effect estimates for the four traits. Our set did not include an inbred contributing effects that significantly increased FD and significantly decreased FA, or vice versa, which was expected given the size of our set and the strength of positive correlation between FD and FA (0.88 ≥ r_p_ ≥ 0.74). A larger population size would be required to adequately search for inbreds with divergent FD and FA effects. Instances of inbreds with significant effects increasing FD or FA while decreasing SD were not observed either, though additional data may show this is the case for PHG39. The contribution of differences in maturity, density response, and pleiotropy to this relationship are unclear at the moment, however. The direction of RA effects among the lines does not appear to be associated with those for FD, FA, or SD, suggesting the genetic control of this trait may be relatively independent by comparison. There was no apparent relationship between the heterotic pool from which a parent was derived and the contribution of positive or negative FD effects in our set of material. This suggests that the reciprocal recurrent selection and pedigree breeding in commercial germplasm has not indirectly resulted in differences in root complexity between the heterotic pools.

### Relationship of root architecture to plant performance

Hammer et al. (2009) simulated the relationship of the historical maize yield using data from multiple decades of corn improvement and some field experiments. The authors noted that the median predicted yield at higher densities increased significantly with narrower root angles across a variety of soil types, but only in the growing seasons that were wettest at the time of planting. However, root system architecture was more important for increasing yield at high density than canopy architecture. The gain in performance in the model was related to narrower root angles being associated with deeper root systems that could capitalize on additional water availability for biomass production. Hammer et al. (2009) estimated that modern maize requires about 270 mm more water per season than a century ago, due to the 6 Mg ha^−1^ historical increase in maize grain yield. While rooting depth was the critical architectural factor in their simulation, the authors noted that enhanced water extraction capacity could also explain the improvement in performance at higher density. Changes in root architecture, but not biomass partitioning, have been implicated in improved performance of maize for yield under drought stress (Ribaut et al. 2009). There are few studies that directly measure partitioning of root biomass under field conditions, but a synthesis of a variety of studies suggest that, under normal conditions, root biomass is maximized around anthesis, and the ratio of root to stover (shoot) biomass gradually decreases from about 2:3 at emergence to 1:3 at flowering to 1:5 at physiological maturity (Amos and Walters 2006).

## Future work

From the study of root complexity in the Ex-PVP material, there are several relationships of interest for further investigation. Inbreds and hybrids with high FD should perform better under drought conditions, due to more efficient capture of water for a given amount of root biomass. However, if root complexity is highly correlated with root biomass, increased complexity might be disadvantageous due to the importance of having a high allocation of biomass to the shoot for good performance under drought (Bänziger et al. 2000). Narrow root angles are also expected to be of assistance in water uptake under drought, as the root biomass may be directed deeper into the soil, where more remaining moisture would be available. Narrow root angles may also be advantageous at increased planting densities, where plants compete for lateral soil space. Increased root complexity may be expected to increase the strength of plant anchorage, which could reduce the frequency of lodging. The relationship between root complexity and fertility is more complex, since the most advantageous type of root structure is dependent on the most limiting type of nutrient(s) and local distribution. If root complexity is not predictive of nutrient uptake, the relationship of root structure to grain yield might be more related to the ability to adapt root growth to the environment before flowering. The relationship of root complexity and topology to performance in the context of plant density stress is of great interest, as increased yield at higher plant densities has been a major element of commercial hybrid improvement over time. Below ground, increased planting density increases the proximity of root systems, thereby increasing plant competition for nutrients and water.

## Additional file

Additional file 1. In the Supplementary Material and Results Section information characterizing the used ex-PVP inbreds and the weather conditions during field experimentation is given and additional results of the quantitative genetic analysis are presented.
