# Supplementary material for: Characterization of mature maize (Zea mays L.) root system architecture and complexity in a diverse set of Ex-PVP inbreds and hybrids
Source: Springerplus. 2015 Aug 18;4:424. doi: 10.1186/s40064-015-1187-0 (PMC4537857; doi:10.1186/s40064-015-1187-0)
Supplement: Supplementary file 1 — In the Supplementary Material and Results Section information characterizing the used ex-PVP inbreds and the weather conditions during field experimentation is given and additional results of the quantitative genetic analysis are presented. [file 40064_2015_1187_MOESM1_ESM.docx]

**Table S1.** Pedigree and background of ex-PVP inbreds used as parents in this study.

| Line^1^ | Pedigree^2^ | Background^3^ | PVP Certificate No. / Date |
| --- | --- | --- | --- |
| B73 | Iowa Stiff Stalk Synthetic Cycle 5 | Stiff Stalk C5 | Not applicable |
| LH1 | B37 (B37 x Holden line 644) | Stiff Stalk C0/Broadbase | 7600047 / July 19, 1977 |
| PHJ40 | PHB09 x PHB36 | Stiff Stalk C0/Broadbase | 8600133 / Mar 31, 1987 |
| PHG39 | PHA33GB4 x PHA34CB4 | Stiff Stalk C0/Maiz Amargo | 8300115 / July 25, 1984 |
| LH82 | LH7 x Holden line 610 | Broadbase/Minnesota 13 | 8500037 / July 26, 1985 |
| LH123 | PHI hybrid 3535 | Broadbase | 8400030 / Feb 22, 1985 |
| PHZ51 | PH814 x PH848 | Lancaster/Broadbase | 8600132 / Mar 31, 1987 |
| PHG84 | PH595 x PH848 | Oh07-Midland/Broadbase | 8600130 / Jan 30, 1987 |
| PHG35 | PH595 x PHG3BD2 | Oh07 Midland/Iodent | 8300140 / Oct 26, 1984 |
| PHG47 | PH041 x MKSDTE Cycle 10 | Oh43/Broadbase | 8600131 / Jan 30, 1987 |
| PH207 | PHG3BD2 x PHG3RZ1 | Iodent | 8300144 / Dec 21, 1984 |
| Mo17 | C103 x C.I. 187-2 | Lancaster | Not applicable |

^1^ Public lines were developed by Iowa State University (B73) and University of Missouri (Mo17). Proprietary lines were developed by Pioneer Hi-Bred Intl (PH) and Holden's Foundation Seeds, Inc. (LH). The proprietary lines were originally registered and made available upon expiration by U.S. Plant Variety Protection.

^2^ Pedigree derivation as described in breeding history by originator.

^3^ Background classification is similarity or grouping by pedigree. “Broadbase” indicates genetically diverse and/or uncertain background. Separation by '/' indicates pedigree consisting of a conglomerate of backgrounds.

**Table S2** Temperature (^◦^F), precipitation, dryness, evaporation, and water deficit in the 2007 and 2010 Urbana, IL. environments.

|  | Max Air Temp^1^ | Min Air Temp^2^ | Avg Temp^3^ |  | Inches of  Rain Fall | Total Evap^4^ | Water Deficit^5^ | Days Dry^6^ |
| --- | --- | --- | --- | --- | --- | --- | --- | --- |
|  |  |  |  | **2007** |  |  |  |  |
| April | 88.7 | 20.8 | 51.4 |  | 2.13 | 4.11 | -1.98 | 9 |
| May | 91.3 | 37.4 | 69.3 |  | 1.54 | 5.96 | -4.42 | 17 |
| June | 96.2 | 48 | 74.8 |  | 5.66 | 6.59 | -0.93 | 13 |
| July | 91.3 | 54.4 | 74.3 |  | 3.82 | 7.03 | -3.21 | 11 |
| August | 96.5 | 58.4 | 77.8 |  | 0.87 | 6.32 | -5.45 | 21 |
| September | 96.4 | 41.1 | 70.4 |  | 2.27 | 5.09 | -2.82 | 16 |
| May to July^7^ |  | Average: | 72.8 | Sum: | 11.02 | 19.58 | -8.56 | 41 |
|  |  |  |  | **2010** |  |  |  |  |
| April | 84.9 | 32.3 | 59.2 |  | 1.91 | 4.77 | -2.86 | 14 |
| May | 92 | 35.1 | 65 |  | 3.09 | 4.42 | -1.33 | 8 |
| June | 91.8 | 57.8 | 74.8 |  | 7.82 | 4.96 | 2.86 | 3 |
| July | 94.5 | 55.8 | 77.3 |  | 3.57 | 5.07 | -1.50 | 8 |
| August | 96.3 | 54 | 77.2 |  | 1.58 | 5.17 | -3.59 | 10 |
| September | 94.3 | 45.5 | 67.5 |  | 3.02 | 4.07 | -1.05 | 7 |
| May to July |  | Average: | 72.4 | Sum: | 14.48 | 14.45 | 0.03 | 19 |

^1^ Maximum air temperature; ^2^ Minimum air temperature; ^3^ Average air temperature;  ^4^ Total moisture evaporated in inches; ^5^ Inches of rainfall – inches of evaporation; ^6^ number of days in a month with no rain ( < 0.1 inches); ^7^ The experiment was planted at the beginning of May in both years. Root growth is expected to be completed around flowering, which occurred in July;

Data provided by the Illinois State Water Survey (ISWS) located in Champaign and Peoria, Illinois, and on the web at www.isws.illinois.edu.

**Table S3** Repeatability estimates, and 90% confidence limits for root complexity and architecture traits among 12 inbreds and their 66 F_1_ crosses.

| Trait ^1^ | Generation | LL ^2^ | Repeatability ^3^ | UL^4^ |
| --- | --- | --- | --- | --- |
| FD | F_1_ | 0.48 | 0.67 | 0.73 |
|  | Inbred | 0.72 | 0.89 | 0.95 |
| FA | F_1_ | 0.59 | 0.74 | 0.79 |
|  | Inbred | 0.65 | 0.87 | 0.93 |
| RA | F_1_ | 0.65 | 0.78 | 0.82 |
|  | Inbred | 0.72 | 0.89 | 0.94 |
| SD | F_1_ | 0.14 | 0.39 | 0.54 |
|  | Inbred | 0.00 | 0.50 | 0.76 |

^1^ FDV, fractal dimension; FA, fractal abundance; RA, root angle; SD, stalk diameter in pixels.

^2^ Lower limit of 90% confidence interval on repeatability calculated as 1 – ((MS ENT/MS GxE)*F distribution inverse(.1, Entry degrees of freedom, GxE degrees of freedom) )^-1

^3^ Repeatability was calculated as $\frac{\delta_{g}^{2}}{\delta_{g}^{2}+\frac{\delta_{gxe}^{2}}{r}+\frac{\delta_{err}^{2}}{re}}$ where genetic variance, $\delta_{g}^{2}$, was calculated as (MS ENT – MS GxE)/re, variance of the interaction of genotypes with the environment was calculated as (MS GxE – MS ERR)/r, r is the number of replications, and e is the number of environments. The model used to obtain the mean squares for repeatability estimates, Y = Environment + Rep(Environment) + Entry + Entry*Environment + Residual Error, does not take into account the covariance between the hybrids due to common parents. Degrees of freedom for the entries (F_1_s/inbreds), environment, entry by environment interaction (F_1_/inbred), replications nested within the environment, and error degrees of freedom (F_1_/inbred) are (65/11), 1, (62/11), 4, and (253/ 44), respectively.

^4^ Upper limit of 90% confidence interval on repeatability calculated as 1 – ((MS ENT/MS GxE)*F distribution inverse(.9, Entry degrees of freedom, GxE degrees of freedom) )^-1

**Table S4** Order of magnitude obtained by studies applying the box counting method to determine the fractal dimension of roots based on root images.

| Species | Stage [DAP] | Box size range [mm] | Order of Magnitude^1^ | Study^2^ |
| --- | --- | --- | --- | --- |
|  |  |  |  |  |
| Wheat | 126 | 0.40 – 21.00 | 1.72 | Tatsumi et al. (1989) |
|  |  | 2.00 – 10.00 | 0.70 | Manschadi et al. (2008) |
|  |  |  |  |  |
| Rye | 126 | 0.40 – 20.00 | 1.70 | Tatsumi et al. (1989) |
|  |  |  |  |  |
| Rice |  |  | 2.50 | Wang et al. (2009) |
|  |  |  |  |  |
| Sorghum | 126 | 0.60 – 16.20 | 1.43 | Tatsumi et al. (1989) |
|  |  |  | 0.60 | Masi and Maranville (1998) |
|  |  |  |  |  |
| Maize | 30 | 0.70 – 19.9 | 1.45 | Tatsumi et al. (1989) |
|  |  |  | 0.60 | Eghball et al. (1993) |
|  | 80 | 1pix – 512pix | 2.71 | *This study* |
|  |  |  |  |  |
| Millet | 30 | 0.65 – 18.80 | 1.46 | Tatsumi et al. (1989) |
|  |  |  |  |  |
| Garden Pea | 58 | 0.28 – 6.60 | 1.37 | Tatsumi et al. (1989) |
|  |  |  |  |  |
| Peanut | 58 | 0.30 – 6.70 | 1.34 | Tatsumi et al. (1989) |
|  |  |  |  |  |
| *Betula populifolia* |  | 59 pix/cm – 59×10pix/cm | 1.00 | Berntson (1994) |
| *Betula alleghaminsis* |  | 59 pix/cm – 59×10pix/cm | 1.00 | Berntson (1994) |

^1^ Order of Magnitude was calculated as $\log_{10} {(s}_{max}/s_{min})$, with *s_ma_*_x_ and *s_min_* are the largest and smallest box size used, respectively.

^2^ Berntson, G.M., 1994 Root systems and fractals: How reliable are calculations of fractal dimensions? Annals of Botany **73**:281-284.

Eghball, B., J.R. Settimi, J.W. Maranville, and A.M. Parkhurst, 1993. Fractal analysis for morphological description of corn roots under nitrogen stress. Agron J. **85**:287-289.

Masi, C. E. A., and J. W. Maranville, 1998 Evaluation of sorghum root branching using fractals. Journal of Agricultural Science, Cambridge **131:** 259-265.

Manchadi, M.M., G.L. Hammer, J.T. Christopher, and P. deVoil, 2008. [Genotypic variation in seedling root architectural traits and implications for drought adaptation in wheat (*Triticum aestivum* L.)](http://www.springerlink.com/content/6p6m4187574p8317/). Plant and Soil **303**:115-129.

Tatsumi, J., A. Yamauchi, and Y. Kono, 1989 Fractal analysis of plant root systems. Ann Bot **64:** 499-503.

Wang, H., J. Siopongco, L. J. Wade, and A. Yamauchi, 2009 Fractal analysis on root systems of rice plants in response to drought stress. Environmental and Experimental Botany **65:** 338-344.

**
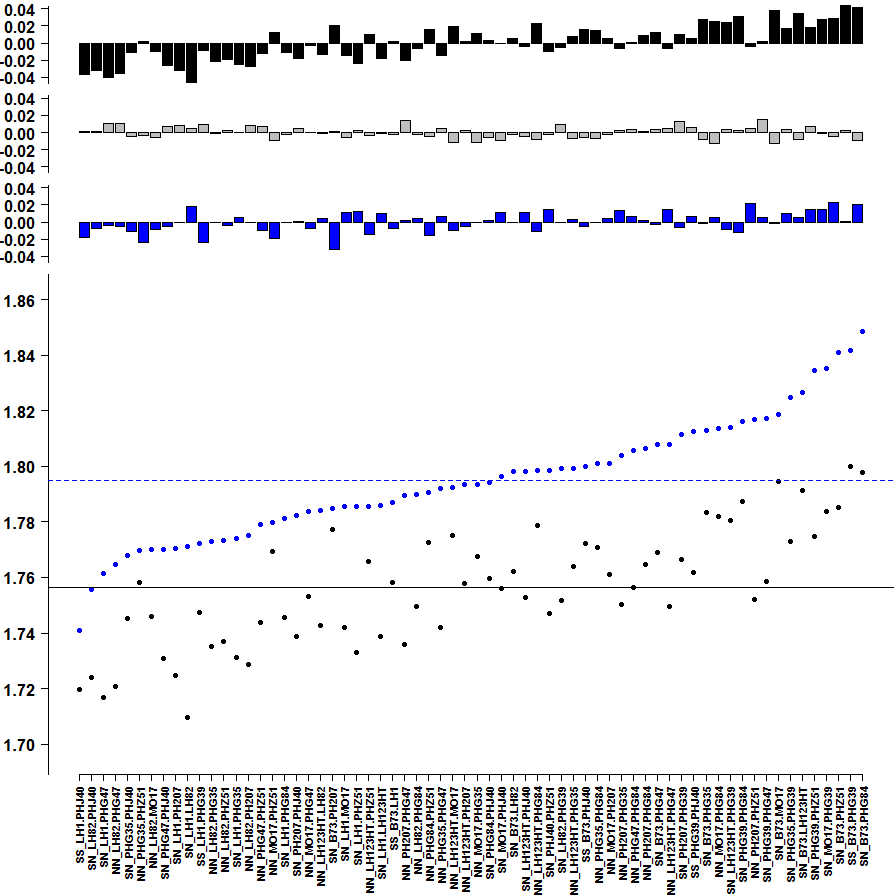
**

**Fig. S1** Chart of genetic effects and mid-parent values for the FD trait among the experimental F_1_s, sorted from lowest to highest performance. The Y-axis scale is in the dimensionless units of the FD trait. Black, grey, and blue bars are the magnitude of the additive, GCA, and SCA effects for the hybrids, respectively. Blue and black dots are F_1_ least square means and mid-parent values, respectively. The dashed blue line and solid black line are the means of the F_1_ and inbred generations. SS, SN, and NN labels affixed to the hybrid names indicate stiff stalk x stiff stalk, stiff stalk x non-stiff stalk (or non-stiff stalk x stiff stalk), and non-stiff stalk by non-stiff stalk hybrid combinations. Individual effect significances are not reported. Overall significance of additive effects *per se*, GCA, SCA, and total heterosis (F_1_ mean – Inbred mean) is provided in the text of the article.


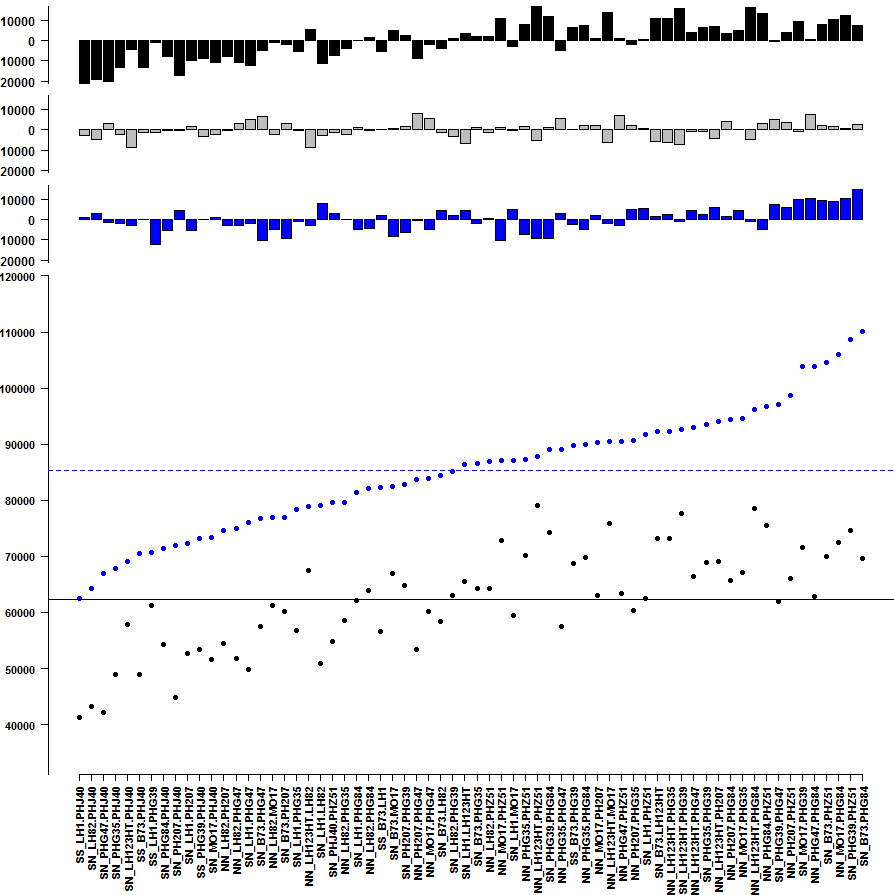


**Fig. S2** Chart of genetic effects and mid-parent values for the FA trait among the experimental F1s, sorted from lowest to highest performance. The Y-axis scale is in the dimensionless units of the FA trait. Black, grey, and blue bars are the magnitude of the additive, GCA, and SCA effects for the hybrids, respectively. Blue and black dots are F1 least square means and mid-parent values, respectively. The dashed blue line and solid black line are the means of the F1 and inbred generations. SS, SN, and NN labels affixed to the hybrid names indicate stiff stalk x stiff stalk, stiff stalk x non-stiff stalk (or non-stiff stalk x stiff stalk), and non-stiff stalk by non-stiff stalk hybrid combinations. Individual effect significances are not reported. Overall significance of additive effects per se, GCA, SCA, and total heterosis (F1 mean – Inbred mean) is provided in the text of the article.


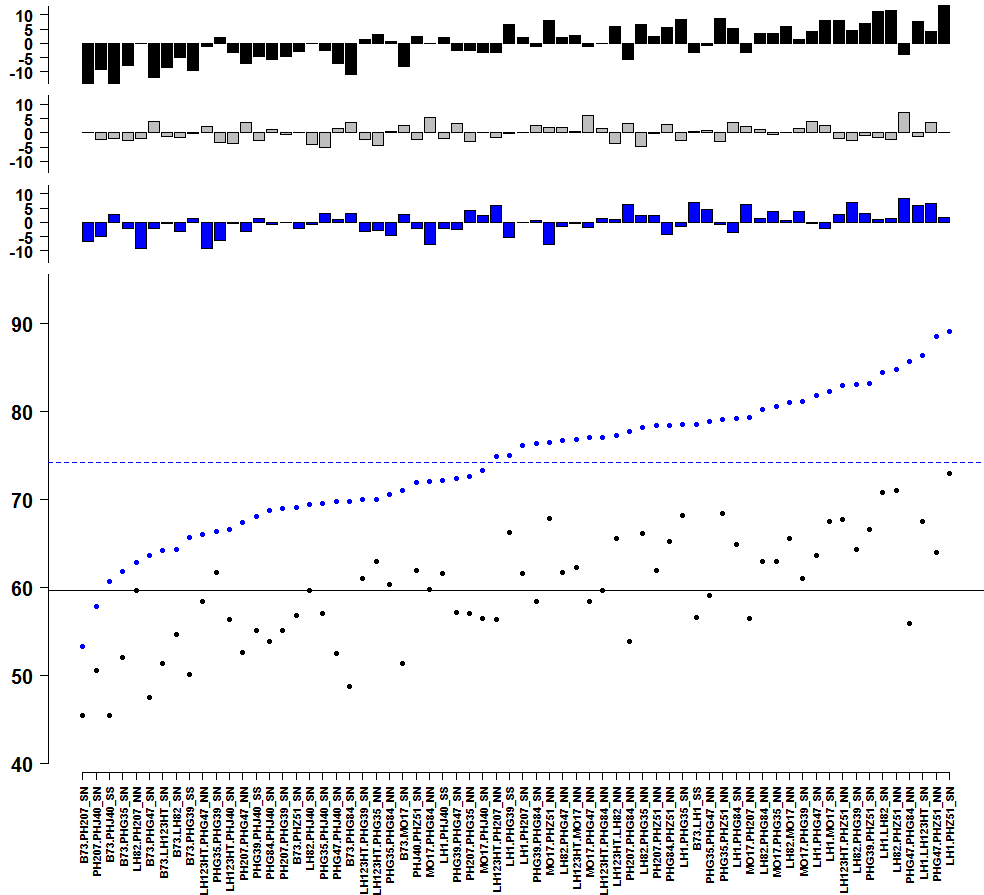


**Fig. S3** Chart of genetic effects and mid-parent values for the RA trait among the experimental F_1_s, sorted from lowest to highest performance. The Y-axis scale is in degrees. Black, grey, and blue bars are the magnitude of the additive, GCA, and SCA effects for the hybrids, respectively. Blue and black dots are F_1_ least square means and mid-parent values, respectively. The dashed blue line and solid black line are the means of the F_1_ and inbred generations. SS, SN, and NN labels affixed to the hybrid names indicate stiff stalk x stiff stalk, stiff stalk x non-stiff stalk (or non-stiff stalk x stiff stalk), and non-stiff stalk by non-stiff stalk hybrid combinations. Individual effect significances are not reported. Overall significance of additive effects *per se*, GCA, SCA, and total heterosis (F_1_ mean – Inbred mean) is provided in the text of the article.


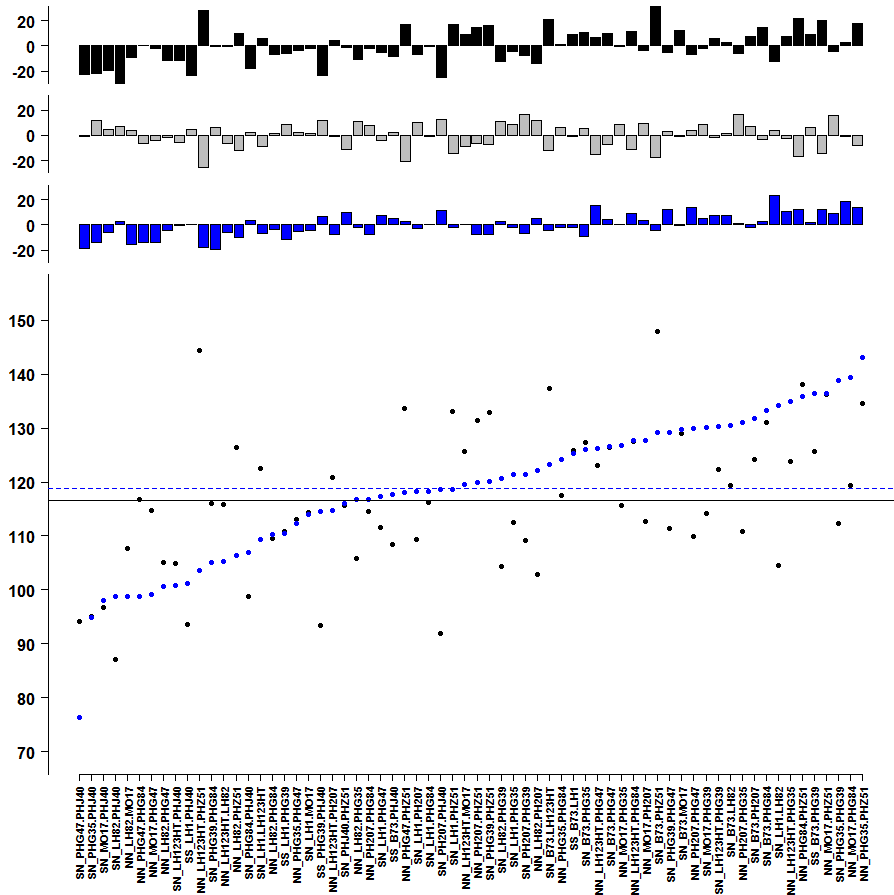


**Fig. S4** Chart of genetic effects and mid-parent values for the SD trait among the experimental F_1_s, sorted from lowest to highest performance. The Y-axis scale width in pixels. Black, grey, and blue bars are the magnitude of the additive, GCA, and SCA effects for the hybrids, respectively. Blue and black dots are F_1_ least square means and mid-parent values, respectively. The dashed blue line and solid black line are the means of the F_1_ and inbred generations. SS, SN, and NN labels affixed to the hybrid names indicate stiff stalk x stiff stalk, stiff stalk x non-stiff stalk (or non-stiff stalk x stiff stalk), and non-stiff stalk by non-stiff stalk hybrid combinations. Individual effect significances are not reported. Overall significance of additive effects *per se*, GCA, SCA, and total heterosis (F_1_ mean – Inbred mean) is provided in the text of the article.

|  |  |
| --- | --- |

**Fig. S5** Scatter plot comparing PC1 (plot **A**) and PC2 (plot **B**) loadings obtained from experiments testing 66 F_1_ hybrids under low nitrogen (“No N Added”) and normal (“Normal Fertility”) nitrogen fertilization.
